# Maternal vitamin and iron supplementation and risk of infant leukaemia: a report from the Children's Oncology Group

**DOI:** 10.1038/sj.bjc.6605957

**Published:** 2010-10-26

**Authors:** A M Linabery, S E Puumala, J M Hilden, S M Davies, N A Heerema, M A Roesler, J A Ross

**Affiliations:** 1 Division of Pediatric Epidemiology and Clinical Research, Department of Pediatrics, University of Minnesota, 420 Delaware Street SE, MMC 422, Minneapolis, MN 55455, USA; 2 Peyton Manning Children's Hospital at St Vincent, 8402 Harcourt Road, Suite 603, Indianapolis, IN 46260, USA; 3 Division of Hematology/Oncology, Cincinnati Children's Hospital Medical Center, 3333 Burnet Avenue, CHRF-2323, Cincinnati, OH 45229, USA; 4 Department of Pathology, The Ohio State University, 129 Hamilton Hall, 1645 Neil Avenue, Columbus, OH 43210, USA; 5 University of Minnesota Masonic Cancer Center, 420 Delaware Street SE, MMC 422, Minneapolis, MN 55455, USA

**Keywords:** epidemiology, infants, vitamin, leukaemia

## Abstract

**Background:**

Prenatal supplementation has been inversely associated with childhood, but not with infant, leukaemia.

**Methods:**

Mothers of 443 cases of infant leukaemia diagnosed during 1996–2006 and 324 frequency-matched controls completed interviews. Associations were evaluated by unconditional logistic regression.

**Results:**

We observed no associations between prenatal vitamin (odds ratio (OR)=0.79, 95% confidence interval (CI): 0.44–1.42) or iron supplementation (OR=1.07, 95% CI: 0.75–1.52) and infant leukaemia after adjustment for race/ethnicity and income. Similar results were observed for leukaemia subtypes analysed separately.

**Conclusion:**

The observed null associations may be attributable to high supplementation rates and/or national fortification programmes.

Leukaemias diagnosed in infants (<12 months) are distinct from leukaemias in older children/adolescents. Proportions of acute lymphoblastic leukaemia (ALL) and acute myeloid leukaemia (AML) cases are more similar among infants (47 *vs* 37%, respectively; Linabery and Ross, 2008) and most cases (ALLs: ∼75%, AMLs: ∼60%) have mixed lineage leukaemia (*MLL*) gene rearrangements in their leukaemia cells (Pui *et al*, 1995; Greaves, 1996). Monozygotic twins with infant leukaemia have high concordance (∼100% *vs* children/adolescents: ∼10% Greaves *et al*, 2003). This observation, coupled with backtracking studies demonstrating that *MLL* translocations are consistently present at birth in infant cases (Gale *et al*, 1997), provides compelling evidence of *in utero* initiation.

Although there are no established infant leukaemia risk factors, prenatal vitamin supplementation has been inversely associated with childhood ALL (see meta-analysis in Milne *et al*, 2010), with one study implicating folic acid (Thompson *et al*, 2001). Sufficient prenatal folic acid significantly reduces congenital abnormalities (MRC Vitamin Study Research Group, 1991). Accordingly, the US Public Health Service recommended ⩾400 *μ*g of daily folate for women of childbearing age in 1992 (Centers for Disease Control, 1992) and the United States and Canada implemented national fortification programmes during 1996–1998 (Food and Drug Administration, 1996; Ray *et al*, 2002b).

Prenatal iron supplementation (30 mg per day) is also recommended (Centers for Disease Control and Prevention, 1998) and has been inconsistently associated with childhood leukaemia (McKinney *et al*, 1999; Thompson *et al*, 2001; Wen *et al*, 2002; Ross *et al*, 2005; Dockerty *et al*, 2007; Kwan *et al*, 2007; Milne *et al*, 2010). In this study, we investigate the effects of prenatal vitamin and iron supplementation on infant leukaemia risk.

## Materials and methods

Study methods have been previously published (Spector *et al*, 2005; Puumala *et al*, 2009, 2010; Johnson *et al*, 2010) and are described briefly below.

### Participant eligibility/identification

#### Cases.

Infants (<12 months) with confirmed acute leukaemia diagnoses during two periods (phase 1: January 1996 to October 2002, phase 2: January 2003 to December 2006) were eligible if they were diagnosed/treated at the US or Canadian Children's Oncology Group (COG) institutions, did not have Down syndrome, had physician approval for contact, had biological mothers who spoke English or Spanish (phase 2), consented to participate, and were available by telephone. Deceased cases were eligible.

#### Controls.

Controls were frequency matched to cases on birth year and location of residence. Controls had biological mothers who spoke English or Spanish, consented to participate, and were available by telephone.

In phase 1, controls were identified through random digit dialing (RDD; Robison and Daigle, 1984). Owing to secular telephone trends (Ross *et al*, 2004), RDD was undesirable for phase 2. Instead, rosters of potential controls, randomly selected on anticipated birth year distribution, were requested from state birth registries. Subjects were randomly selected from 15 state rosters. If mothers refused, replacement subjects were selected until willing participants were identified.

### Data collection

In telephone interviews, mothers were asked whether they consumed vitamin supplements anytime in the year before or during the index pregnancy; in the year before pregnancy; early in but before knowledge of pregnancy; and after knowledge of pregnancy. For each time period, we asked what types of supplements were consumed and whether or not supplements were prescribed by healthcare professionals. Indicator variables were created to assess vitamin use in the periconceptional period (year before pregnancy and early in but before knowledge of pregnancy) and from 1 year before through the index pregnancy. An equivalent set of items concerned iron supplementation exceeding the iron found in multivitamins.

Mothers of cases provided diagnostic information, including results of Southern blot, RT–PCR, fluorescent *in situ* hybridisation, or other cytogenetics testing. Three independent reviewers (SMD, NAH, JMH) evaluated the submitted materials to determine whether there was evidence of *MLL* gene rearrangement (MLL+, *n*=228), evidence of no rearrangement (MLL−, *n*=146), or insufficient evidence (*n*=69).

### Statistical methods

Unconditional logistic regression (SAS 9.2, SAS Institute Inc., Cary, NC, USA) was performed to quantify associations between maternal supplement consumption and acute leukaemia among combined cases, and among ALL, AML, MLL+, and MLL− cases analysed separately. Odds ratios (ORs) and 95% confidence intervals (CIs) were produced. Potential confounders selected *a priori* are listed in Table 1. Variables were retained in multivariable models if they substantially (⩾10%) changed ln(OR) estimates, including maternal race/ethnicity (white, black, Hispanic or other) and household income in the child's birth year (⩽$30 000, $30 001–75 000 or >$75 000). Adjustment for matching factors (birth year and region of residence) did not materially alter point estimates; hence, they were not included in the final models.

Institutional review boards at the University of Minnesota, participating COG institutions, and states providing birth certificate data (as needed) approved the study.

## Results

Overall, mothers of 443 cases (*n*_ALL_=264, *n*_AML_=172) and 324 controls participated in this study. In phase 1, 240 eligible case (69%) and 255 eligible control (59%) mothers completed interviews (Spector *et al*, 2005). One control was excluded from analysis because the child was found to have Down syndrome during the interview. In phase 2, 345 potential cases were identified through COG institutions, 240 were enroled, and 203 mothers completed interviews (59%) (Johnson *et al*, 2010). We identified 267 potential birth certificate controls, of which 70 completed and 1 partially completed the interviews (27%) (Puumala *et al*, 2009). Controls from each phase were similar enough on important demographic factors to merge them in the current analysis (Puumala *et al*, 2009).

Cases and controls were similar in infant gender, birth weight, and length of gestation, and in maternal age at index child's birth, previous foetal loss, and smoking during pregnancy (Table 1). More case mothers had <high school diploma (34 *vs* 28% of control mothers), were non-white (24 *vs* 15%), had a lower income (36 *vs* 30% earning ⩽$30 000), experienced morning sickness (71 *vs* 63%), and reported no alcohol consumption during the index pregnancy (86 *vs* 79%).

Notably, 91% of case and 94% of control mothers reported vitamin use in the year before and/or during the index pregnancy. After adjustment for race/ethnicity and income, there were no associations between vitamin use in the year before and/or during pregnancy (OR=0.79, 95% CI: 0.44–1.42), in the periconceptional period (OR=0.89, 95% CI: 0.64–1.24), after knowledge of pregnancy (OR=0.78, 95% CI: 0.48–1.28), or use over all periods (OR=0.84, 95% CI: 0.62–1.14) and infant leukaemia (Table 2). Restricting exposure to use only after knowledge of pregnancy generated comparable results with those described above (data not shown). Similar results were observed among ALL and AML cases analysed separately (Table 2); ORs for ALL were consistently <1.00, whereas ORs for AML fluctuated around the null. Stratification on *MLL* translocation status did not provide notable findings (data not shown), with the exception of ALL MLL+ cases, in whom reduced risk was suggested for exposure throughout the periconceptional/prenatal periods (OR=0.66, 95% CI: 0.44–1.00).

A number of stratifications were performed to assess the robustness of results. Across the two phases, equal proportions of case mothers reported vitamin use (91% reported any use in both phases) and there was no evidence of heterogeneity of the ORs (data not shown). There was also no detectable heterogeneity on stratification by folic acid fortification period (before, during, and after fortification) or region of residence (six US regions and Canada), although there was limited power to detect differences, given smaller cell counts (data not shown).

There was no evidence of an effect of iron supplementation anytime in the year before and/or during pregnancy (OR=1.07, 95% CI: 0.75–1.52), in the periconceptional period (OR=1.23, 95% CI: 0.63–2.38), after knowledge of pregnancy (OR=1.06, 95% CI: 0.74–1.53), or use in all periods (OR=2.54, 95% CI: 0.69–9.39) after accounting for race/ethnicity and income (Table 2). Among those who reported iron supplementation, there was no association between prescription use and infant leukaemia (data not shown). Further analysis by leukaemic subtype (ALL *vs* AML, MLL+ *vs* MLL−) did not yield significant findings (Table 2).

## Discussion

We found no evidence supporting associations between periconceptional/prenatal vitamin or iron supplementation and infant leukaemia, either overall or for specific aetiological time periods. These results are consistent with other reports regarding infant leukaemia (Wen *et al*, 2002; Pombo-de-Oliveira and Koifman, 2006). In contrast, most childhood ALL studies have suggested inverse associations with prenatal vitamin supplementation (with or without iron) and/or with specific periconceptional/prenatal periods (Sarasua and Savitz, 1994; Thompson *et al*, 2001; Wen *et al*, 2002; Ross *et al*, 2005; Dockerty *et al*, 2007; Schuz *et al*, 2007; Milne *et al*, 2010). However, no associations have been reported for childhood AML (Robison *et al*, 1989; Ross *et al*, 2005; Schuz *et al*, 2007).

The reduced odds observed in ALL MLL+ cases may warrant additional study, as folate deficiency is correlated with increased DNA double-strand breaks in blood and bone marrow (Blount *et al*, 1997), and double-strand breaks precede *MLL* translocations (Reichel *et al*, 1998). Further, ALL MLL+ case mothers were expected to recall exposures similar to mothers in other subgroups.

Prenatal iron supplementation may indicate low iron levels or anaemia, and maternal anaemia has been associated with childhood leukaemia (Petridou *et al*, 1997; Roman *et al*, 1997, 2005). These observations, along with inverse associations between prenatal iron supplementation and childhood leukaemia reported by some (Wen *et al*, 2002; Kwan *et al*, 2007), suggest that iron deficiency may be related to childhood leukaemia. Conversely, there were no associations between prenatal iron supplementation or gestational anaemia, as documented in medical records, and infant leukaemia in phase 1 of this study (Peters *et al*, 2006), which aligns with our results.

This study has strengths and limitations. It comprises the largest study of infant leukaemia conducted to date; previous investigations included 136 and 202 cases (Alexander *et al*, 2001; Pombo-de-Oliveira and Koifman, 2006). Further, use of the COG registry in case ascertainment results in a nearly population-based study population, as COG institutions see ∼100% of leukaemia cases aged 0–4 years (Ross *et al*, 1996).

Differential recall is a concern, as case mothers may exert extra effort to accurately recall exposures. Results of validation studies (Mackenzie and Lippman, 1989; Drews *et al*, 1990; Burton *et al*, 2001) suggest that, although accuracy of maternal supplementation recall may vary slightly by case–control status and time period of assessment, resulting effect estimates and aetiological inferences are comparable. In this study, the early age of leukaemia onset limited the recall period.

There are other potential sources of misclassification. Most mothers reported taking multi- or prenatal vitamins containing many nutrients, thereby precluding identification of aetiologically relevant component(s). We restricted our analysis and found that 98% consumed vitamins with folic acid; ORs were nearly identical to those in Table 2 (data not shown). We were unable to assess total dietary folate or iron intake because of the limited food frequency questionnaire employed in the interview. However, dietary data from a representative sample of non-pregnant US adults surveyed after fortification suggest that supplement use may be a useful measure of variation in folic acid exposure (Yeung *et al*, 2008).

Further, we might only expect to observe an association in the presence of folate deficiency (Robien and Ulrich, 2003); however, US and Canadian fortification programmes increased folic acid intake among women of childbearing age early in the study period (Centers for Disease Control and Prevention, 2000; Honein *et al*, 2001; Ray *et al*, 2002a, 2002b). Of note, neuroblastoma and Wilms tumour incidence rates decreased after Canadian fortification, but no association was observed with infant ALL (French *et al*, 2003; Grupp *et al*, 2010). The high level of supplementation in our study also limited statistical power.

Differential response rates across cases and controls may indicate selection bias, as study participation (Law *et al*, 2002) and prenatal vitamin use (Langley-Evans and Langley-Evans, 2002; Williams *et al*, 2003) may vary by SES, race, and/or educational attainment. Our prior analysis showed that RDD and birth certificate controls were similar to one another; however, control participants differed from the US population and from non-participants on relevant demographic factors (Puumala *et al*, 2009). In this study, we adjusted for maternal race/ethnicity and income, but acknowledge that residual confounding may remain.

In contrast to previous childhood leukaemia studies, we did not observe a prenatal vitamin–infant leukaemia association. This may be attributable to high folic acid supplementation rates, including personal vitamin use and national fortification programs implemented during the study period. Similarly, we did not observe an association with iron supplmentation above that found in multi- or prenatal vitamins.

## Figures and Tables

**Table 1:** Selected characteristics of 443 infant leukaemia cases and 324 controls

	**Controls**	**Combined cases**		
	***N* (%)**	***N* (%)**	**OR**	**95% CI**
*Infant characteristics*				
Gender				
Male	156 (48.2)	218 (49.2)	1.00	
Female	168 (51.9)	225 (50.8)	0.96	0.72–1.28
Birth weight				
<2500 g	17 (5.3)	23 (5.2)	0.99	0.52–1.90
2500–4000 g	258 (79.6)	351 (79.2)	1.00	
>4000 g	49 (15.1)	69 (15.6)	1.04	0.69–1.54
Length of gestation				
<38 weeks	35 (10.8)	55 (12.4)	1.17	0.75–1.84
38–42 weeks	288 (88.9)	387 (87.4)	1.00	
>42 weeks	1 (0.3)	1 (0.2)	0.74	0.05–11.95
				
*Maternal characteristics*				
Age at index child's birth				
<35 years	265 (82.0)	372 (84.2)	1.00	
⩾35 years	58 (18.0)	70 (15.8)	0.86	0.59–1.26
Previous foetal loss				
None	241 (74.4)	337 (76.1)	1.00	
1	64 (19.8)	76 (17.2)	0.85	0.59–1.23
⩾2	19 (5.9)	30 (6.8)	1.13	0.62–2.05
Educational attainment				
<High school graduate	91 (28.2)	149 (33.7)	1.47	1.02–2.11
Some post-high school	112 (34.7)	125 (28.3)	1.00	
College graduate	120 (37.2)	168 (38.0)	1.25	0.89–1.77
Race/Ethnicity				
White	273 (84.5)	334 (75.6)	1.00	
African-American	18 (5.6)	18 (4.1)	0.82	0.42–1.60
Hispanic	15 (4.6)	55 (12.4)	3.00	1.66–5.42
Other	17 (5.3)	35 (7.9)	1.68	0.92–3.07
Household income				
⩽$30 000	95 (29.6)	157 (35.8)	1.27	0.91–1.77
$30 001–75 000	145 (45.2)	189 (43.1)	1.00	
>$75 000	81 (25.2)	93 (21.2)	0.88	0.61–1.27
Morning sickness				
No	120 (37.0)	128 (28.9)	1.00	
Yes	204 (63.0)	315 (71.1)	1.45	1.07–1.96
Smoking during pregnancy				
No	258 (79.9)	368 (83.3)	1.00	
Yes	65 (20.1)	74 (16.7)	0.80	0.55–1.15
Drinking during pregnancy				
No	254 (78.6)	377 (85.7)	1.00	
Yes	69 (21.4)	63 (14.3)	0.62	0.42–0.90

Abbreviations: 95% CI=95% confidence interval; OR=odds ratio.

**Table 2:** Association of vitamin use and infant leukaemia

	**Controls**	**Combined cases**			**ALL**			**AML**		
	***N***	***N***	**OR** a	**95% CI**	***N***	**OR** a	**95% CI**	***N***	**OR** a	**95% CI**
*Prenatal vitamins*										
Any prenatal vitamin consumption										
No	19	40	1.00		28	1.00		11	1.00	
Yes	303	402	0.79	0.44–1.42	235	0.63	0.34–1.18	161	1.20	0.53–2.75
Periconceptional consumption										
No	100	162	1.00		104	1.00		56	1.00	
Yes	222	280	0.89	0.64–1.24	159	0.77	0.54–1.11	116	1.05	0.68–1.61
Consumption during pregnancy, after confirmation of pregnancy										
No	29	58	1.00		39	1.00		18	1.00	
Yes	293	384	0.78	0.48–1.28	224	0.66	0.39–1.11	154	1.05	0.55–2.04
Consumption in year before and throughout pregnancy										
No	137	222	1.00		137	1.00		83	1.00	
Yes	185	220	0.84	0.62–1.14	126	0.77	0.55–1.09	89	0.88	0.60–1.31
										
*Prenatal iron supplements*										
Any prenatal iron consumption										
No	253	334	1.00		195	1.00		136	1.00	
Yes	70	108	1.07	0.75–1.52	68	1.22	0.82–1.80	36	0.82	0.51–1.33
Periconceptional consumption										
No	308	414	1.00		247	1.00		160	1.00	
Yes	15	28	1.23	0.63–2.38	16	1.30	0.62–2.72	12	1.26	0.55–2.88
Consumption during pregnancy, after confirmation of pregnancy										
No	260	345	1.00		201	1.00		141	1.00	
Yes	63	97	1.06	0.74–1.53	62	1.22	0.81–1.84	31	0.77	0.46–1.27
Consumption in year before and throughout pregnancy										
No	320	431	1.00		256	1.00		168	1.00	
Yes	3	11	2.54	0.69–9.39	7	3.04	0.77–12.03	4	1.72	0.33–9.06

Abbreviations: 95% CI=95% confidence interval; ALL=acute lymphoblastic leukaemia; AML=acute myeloid leukaemia; OR=odds ratio.

a ORs adjusted for maternal race and household income.
